# People versus machines in the UK: Minimum wages, labor reallocation and automatable jobs

**DOI:** 10.1371/journal.pone.0224789

**Published:** 2019-12-02

**Authors:** Grace Lordan

**Affiliations:** Department of Psychological and Behavioural Science, The London School of Economics and Political Science, London, United Kingdom; TED University, TURKEY

## Abstract

This study follows the Lordan and Neumark (2018) analysis for the US, and examines whether minimum wage increases affect employment opportunities in automatable jobs in the UK for low-skilled low-wage workers. Overall, I find that increasing the minimum wage decreases the share of automatable employment held by low-skilled low-wage workers, and increases the likelihood that workers in automatable jobs become dis-employed. On aggregate the effect size is modest, but I also provide evidence that these effects are larger in more recent years. The study also highlights significant heterogeneity by industry and demographic group, including more substantive adverse effects for older low-skilled workers in manufacturing, as well as effects at the intensive margin.

## Introduction

There are no shortage of papers that consider the effects of the minimum wage in the UK. In general, these studies have focused on potential changes to employment opportunities for the low skilled, and the majority suggest that overall employment effects are minimal [1–2]. However, there is evidence that specific sub groups do lose out on employment opportunities when the minimum wage increases. This underlines the importance of considering differential effects at the individual level to understand completely who the winners and losers are. Specifically, those that lose are part-time women [3–4], part-time workers in general [3], service industry employees [5] and care home workers [6]. In addition, there is evidence of a significant amount of job switching for low-paid workers after minimum wage increases, which has associated costs [7].

Outside employment effects employers can respond in a number of other ways to increases in the minimum wage. For example, they may alter job amenities [8] or compress wages [9–10]. A recent paper by Lordan and Neumark [11] explores whether minimum wage affects the employment possibilities for workers relying on automatable employment indirectly by considering if individuals in automatable jobs are more likely to lose their jobs, following minimum wage increases in the US. The authors note that the adoption of new technology should also create jobs within firms as they require different types of workers to maintain their new technologies, however these roles will be of higher skill than the ones the technology replaced. A related analysis [12] analysed the susceptibility of low-wage employment to technological substitution. This study also provides evidence that firms may automate routine jobs in response to a minimum wage increase, reducing employment opportunities for workers in routine jobs. Given the current attention being paid to the potential for robots to cause unrecoverable job loss in the academic literature [13–16] the objective of this work is to replicate Lordan and Neumark [1] for the UK. Therefore, the main contribution of this work is to be the first exploration of this kind for the UK. Consistent with Lordan and Neumark, I empirically assess whether there are changes in i) the share of automatable employment ii) the propensity to lose employment in an automatable job iii) the propensity to switch from an automatable job iv) the share of hours that are automatable v) the number of hours worked in automatable jobs following a minimum wage increase.

This work is timely given the UK government has committed to regularly revising their minimum wage policies upwards in line with the median earned wage, with the final commitment being fulfilled in 2020. Therefore, my analysis has a clear and general policy perspective given that it informs of the likelihood of losing low skilled automatable employment shares following minimum wage hikes, as well as highlighting what groups are the most vulnerable in terms of any labor reallocations, in time for the next discussions on future minimum wage increases that go beyond current commitments.

The empirical analysis draws on the Quarterly Labor Force Survey data (QLFS) from 1994–2017. While I follow the Lordan and Neumark analysis as closely as possible, a lack of cross area variation in the UK means that the identification strategies do diverge. In this case, the identification strategy becomes less credible for the shares of employment analysis. However, I address this with additional robustness analysis. Consistent with Lordan and Neumark I distinguish between occupations that are high in automatable tasks by drawing on well-accepted US definitions [17–18]. Overall, I consistently highlight that minimum wage increases decrease the shares of automatable employment following a minimum wage increase. On aggregate, the effects found are modest. For example, I find that a 10% increase in the minimum wage leads to a 0.11 percentage point decrease in the share of automatable jobs done by low-skilled workers. Notably this masks a stronger effect over the last decade and a half which is roughly double in size, implying that the importance of the interaction between the minimum wage and automation has been accelerating. In addition, the aggregate effects mask larger changes experienced by specific groups. For example, manufacturing specific estimates imply that a 10% increase in the minimum wage leads to a 0.29 percentage point decline in the share of automatable employment. I note that the pooled analysis also masks significant heterogeneity by demographic groups. Men and the oldest workers are affected the most, with larger effects also evident for White, low-skilled workers. An analysis of hours also reveals that there are effects at the intensive margin, in addition to the extensive margin.

Similar conclusions to the shares of employment analysis emerge in an additional analysis, which considers the likelihood a low-skilled low-wage worker in automatable employment remains employed in the next period as compared to a low skilled low-wage worker in non-automatable employment following a minimum wage increase. Overall, workers in automatable employment are significantly less likely to keep their job and work fewer hours in the next period, however these differences are small. Once again, the effect size roughly doubles when I consider an analysis window between 2004–2017. I also note that those working in manufacturing, men and the oldest workers experience greater declines (for example a 0.10 percentage point decline for low skilled manufacturing workers in automatable employment older than forty years for every 10% increase in the minimum wage). I also find some robust evidence that low-skilled low-wage workers in automatable employment are more likely to switch jobs to non-automatable work in the next period following a minimum wage increase.

## Methodology

### Low-skilled low-wage individuals

This analysis focuses on low-skilled low-wage individuals. I envisage a labor market that is entirely separable by skill. That is to say low-skilled low-wage individuals cannot–at least without more training which takes time–obtain a high-skill job. Therefore, this analysis may be viewed as focusing on how minimum wage increases cause changes to the type of work available for low-skilled low-wage workers only. This is in line with the minimum wage literature in general which focuses on the effects of increases on those that are most likely to be affected only.

Throughout the analysis I consistently define low-skilled low-wage individuals as those who are working in the lowest-paid occupations, while also having low levels of education. I focus only on those born in the UK to circumvent the fact that immigration flows may cause the composition of this group to fluctuate over time. I calculate income for each occupation in each year, and based on this distribution only include in the analysis those in the bottom income quintile with a GCSE equivalent or less. This intersection is important given that the value of holding a GCSE equivalent or less changed markedly over the long-time window used in this study. However, I note that changing the analysis to focus on those who have a GCSE level qualification or below serves only to increase the estimate effects slightly.

### Measuring automatable employment

The measure of automatable employment follows Lordan and Neumark [1] by drawing on definitions provided by [17–18]. Specifically, routine task intensity in each three-digit occupation is defined as:

$${RTI}_{k}=\ln\left(T_{k}^{R}\right)-\ln\left(T_{k}^{M}\right)-ln(T_{k}^{A})$$ (1)

where *T*_*k*_^*R*^, *T*_*k*_^*M*^, and *T*_*k*_^*A*^ are the levels of routine, manual, and abstract task inputs for occupation *k* measured at the 3-digit level (these levels are defined using variables from versions of the Dictionary of Occupation Titles). Therefore, Eq (1) is increasing in the absolute and relative quantity of tasks that are automatable within occupation *k*. Eq 1 is calculated for three-digit UK Standard Occupation Codes (SOC) 2000 occupation codes, based on standardized responses to these questions (mean 0 and standard deviation of 1) and matched to the QLFS from 1992 to 2017. The coding system in the UK changed twice between 1992–2017. Between 1992- Q1 2002 the QLFS used UK SOC 90. I utilize a cross walk described in (19) to assign each UK SOC 90 code a UK SOC 2000 value. In 2010 there was another minor coding change. I have created a cross walk to assign a UK SOC 2000 code to each UK SOC 2010 in the QLFS. In addition, I draw on additional cross walks [19] to allow me crosswalk the SOC 90 values to the coding system used by the classifications of [17–18]. A job is then defined as automatable if it is in the top tertile of Eq (1) and zero otherwise.

### Automatable jobs

Table 1 provides examples of occupations that are classified as automatable and non-automatable. The jobs that are labelled as automatable are easily substitutable with robotics (for example, assemblers and routine operatives) or computer software (for example, administrative occupations in filing records). That is, the technology is readily available now. In contrast, the jobs that are labelled as non- automatable are much less predictable in terms of their sequence of actions (for example, transport drivers and operatives) and often require contact with clients (for example, personal service occupations).

**Table 1:** Examples of automatable job.

Automatable
Elementary administration occupations
Elementary process plant occupations
Assemblers and Routine Operatives
Food Preparation Trades
Administrative Occupations–Records
Non-Automatable
Transport Drivers and Operatives
Personal Service Occupations NEC
Metal Machining, Fitting and Instrument Making Trades
Sales Related Occupations
Customer Service Occupations

### Shares of automatable employment analysis

Based on the definition of automatable work given by the top tertile of Eq (1) for each industry *i*, within each area *a* (defined as government office regions in the UK), in year *t*, I calculate an automatable employment share as:

$${RSH}_{iat}=(\sum_{k=1}^{K}(L_{iat})∙1[{RTI}_{k}>{RTI}^{P66}])(\sum_{k=1}^{K}L_{iat}){)}^{-1}$$ (2)

In Eq (2), ***L_iat_*** is equal to total employment in industry *i* in government area *a* at time *t*. 1[.] takes the value of one if an occupation is in the top third of the employment-weighted distribution of *RTI* across occupations, using only low-skilled workers and those in the bottom quintile of the income distribution (low-wage) as the population sample, and is zero otherwise. The numerator is the share of automatable low-skill low-wage employment in a particular industry, area, and year, and the denominator is total low-skilled low-wage employment in that industry, location, and year. The data I draw on is the QLFS. The QLFS is the main survey of individual economic activity in the UK, and provides the official measure of the national unemployment rate.

Our analysis initially focuses on the following specification

$${RSH}_{iat}=b_{1}LogMW_{t}+A_{a}\lambda ++A_{t}\gamma +I_{i}\varphi +X_{iat}+\varepsilon_{iat}$$ (3)

where *LogMW*_*t*_ denotes the natural log of the minimum wage at time *t* adjusted to 2015 prices (see data section for how minimum wage is defined). Eq (3) includes area (*A*_*a*_) and industry (*I*_*i*_) fixed effects. It also includes area specific time trends (*A*_*t*_*)*. Standard errors are two-way clustered by area crossed by industry and year. *X*_*iat*_ is a set of control variables, which may simultaneously predict the dependent variable, while being correlated with the minimum wage over time. These are: 1) lag of area level unemployment rate. 2) lag of Industry level unemployment rate. 3) lagged area level demographics that vary over time: average age, education, gender. 4) occupation demographics measured at the area/industry/year level. Specifically, these are average age, education, gender. The estimates do not change if lags are replaced with contemporaneous values.

I am specifically interested in *b*_*1*,_ where a negative and significant estimate implies that the share of automatable employment is declining in response to minimum wage increases. This can be interpreted as evidence that minimum wage increases are interacting with automation and causing changes to the type of work available to low-skill low-wage workers in the UK. Or in other words it is changing the type of work available for these workers.

Following Lordan and Neumark [1] I disaggregate these effects across industries and demographic groups, to consider whether there is heterogeneity in the effects found. Consistent with Lordan and Neumark I focus on differences by age sex, ethnicity and one-digit industry code. For ethnicity I look at Whites, Blacks and Asian workers. Here Asian is defined as someone who identifies their ethnic origin as Indian, Pakistani or Bangladeshi. I do not look at other ethnicities given small cell sizes. For age, I look at aged 40 and over, those aged 25 or younger, and the intermediate group aged 26–39. To unpack these differential effects, I create specific measures of task intensity for each subgroup (indexed by *c)*:

$${RSH}_{ciat}=(\sum_{k=1}^{K}(L_{ciat})∙1[{RTI}_{k}>{RTI}^{P66}])(\sum_{k=1}^{K}L_{ciat}){)}^{-1}$$ (4)

In Eq (4) the numerator is the share of automatable employment held by a particular sub-group in a specific industry, area, and year, and the denominator is total employment of a particular subgroup by industry, area, and year. I then estimate Eq (3) separately for each sub-group with *RSH* as defined in Eq (4).

## Data

The main data source for the shares of employment analysis is the QLFS. These data are matched to monthly age-specific data on the minimum wage that was gathered by the author for this work. Following, Lordan and Neumark [1] I allow for a period of adjustment by defining the minimum wage as its average over the current month plus the last 11 months. The minimum wage is measured in 2015 prices. I do not produce sub analysis for the one-digit industries agriculture, forestry and fishing and energy and water as the sample sizes are too small. I create the share of employment variable on a yearly basis, and similarly construct an annual average of the minimum wage variables by calculating its average by industry, area and year.

### Individual-level analysis for automatable employment

I also estimate regressions using individual-level data on low-skilled low-wage individuals. Specifically, I estimate:

$${EMP}_{jiat}=b_{1}({RSH}_{jiat}LogMW_{at})+b_{2}{RSH}_{jiat}+T_{i}S_{s}\gamma +I_{i}\varphi +\varepsilon_{jiat}$$ (5)

In Eq (5) *Emp* is the probability that the *j*^*th*^ person is employed in industry *i*, area *a*, at time *t+*1. It is assigned zero if a person was unemployed in *t*+1. The sample consists of those low-skill low-wage workers employed in period *t*, and either employed or dis-employed in period *t*+1. Eq (5) relates job loss to workers having held a routine job in period *t*, and facing a minimum wage increase with standard errors two-way clustered by area crossed by industry and year. *b*_*1*_ then captures if a person in automatable work is more vulnerable than those in non-automatable work to job loss following a minimum wage increase. I note that Eq (5) speaks to flows out of employment only.

Eq (5) includes area-by-year interactions, to allow for differential time patterns across areas. The identification strategy of Eq (5) is then more conservative as compared with Eq 3 where I relied on area-specific time trends only to pick up time changes. Because of this set of interactions the base minimum wage effect drops out when estimating Eq (5), and identification of *b1* is from variation in the availability of automatable jobs within areas across time. The approach is identical to Lordan and Neumark [1].^.^ All other definitions for Eq (5) remain consistent with Eqs (1) through (3). I unpack heterogeneity in *b*_*1*_ by estimating Eq (5) separately by one-digit industry code, age, ethnicity and gender.

In addition, I estimate separate versions of Eq (5) that replaces the dependent variable with another that equals one if an individual had the same 3-digit occupation code in the year of interview, and zero otherwise (including the dis-employed). In this case, a negative and significant *b*_*1*_ implies that individuals are moving from automatable work towards non-automatable work following a minimum wage increase.

### Individual level analysis data

The individual level analysis relies on the Longitudinal Labour Force Survey (LLFS), which contains a subset of individuals interviewed in the QLFS. In the LLFS respondents are interviewed five times in total–once every quarter for a year. In this analysis the first period is quarter 1 and the second is quarter 5. Therefore, I calculate the probability of still being employed one year later, and the probability of still holding the same occupation with a one-year lag. Minimum wage is then defined at the level that was in effect when the person was interviewed in 2015 prices.

### Analysis of hours

I also consider whether minimum wage increases cause changes to hours in automatable employment like Lordan and Neumark [1] by re-estimating Eq (3) and relating the log of minimum wage to the share of automatable hours. In this case, the numerator is the number of hours worked by low-skill low-wage workers in automatable employment in a particular industry, area and year. The denominator is the total hours worked in an area in a given year.

Finally, I re-estimate Eq (5) with the difference in reported usual weekly hours worked between quarter 5 and quarter 1 by an individual as the dependent variable (this analysis draws on the LLFS instead of the QLFS given I need to know what hours were worked one year in the past as compared to the present). Specifically, I focus on those who are in employment in the two periods, and reported positive hours worked at both of these time points.

## Results

### Effects on employment shares

The results for the employment shares analysis (Eq (4)) are reported in Table 2. From Column (1), the pooled analysis suggests that a 10% increase in the minimum wage leads to a 0.11 percentage point decrease in the share of automatable jobs done by low-skilled workers. This is roughly 1/3 of the overall effect found in Lordan and Neumark [1].

**Table 2:** Shares of employment analysis.

	(1)	(2)	(3)	(4)	(5)	(6)	(7)	(8)
	Pooled	Manufacturing	Construction	Hotels and Restaurants	Transport and Communication	Banking and Finance	P Admin Educ and Health	Other Services
Dependent Variable = Share of Automatable Employment								
MinWage	-0.0112	-0.0288	0.0006	-0.0079	0.0018	-0.0102	-0.0030	-0.0021
	(0.0012)	(0.0027)	(0.0004)	(0.0050)	(0.0015)	(0.0026)	(0.0019)	(0.0014)
N	4320	480	480	480	480	480	480	480

Notes: OLS coefficient estimates are reported, with standard errors in parentheses. Min Wage is in log form. Standard errors are double clustered by area crossed by industry and year. Low-skilled workers are defined as those who have a GCSE equivalent or less. The definition of automatable employment follows Autor and Dorn (2013) and Autor et al. (2015) and is consistent with that used by Lordan and Neumark (2018). A job is classified as automatable at the three-digit occupation code level. The share of automatable employment is calculated by industry, state, and year. All regressions include area fixed effects and area specific time trends. Regressions also include other control variables. These are: 1) Lag of Area level unemployment rate. 2) Lag of Industry level unemployment rate. 3) Lagged Area level demographics that vary over time: average age, education, gender. 4) Occupation demographics measured at the area/industry/year level. Specifically, these are average age, education, gender. All lagged variables relate to one year. I note that the estimates do not change notably (to the third decimal place) if lags are replaced with contemporaneous values.

Consistent with Lordan and Neumark [1] the sub analysis reveals that selected industries are driving the overall effect, in this case manufacturing and banking. In manufacturing, the effects are the largest suggesting that a minimum wage increase of 10% leads to a 0.29 percentage point decrease in the share of automatable jobs done by low-skilled low -wage workers in manufacturing. The respective decline for banking and finance is 0.10 percentage points. Notably, in Lordan and Neumark [1] the finance industries effect is zero, and the overall effect found is driven solely by manufacturing.

Table 3 disaggregates the analysis by age, gender and ethnicity. Considering the age estimates in Table 3, from Column (1), the shares of automatable jobs for low skilled older workers (≥ 40 years old) are the most affected. For example, for workers who are 40 years or older a minimum wage increase of 10% leads to a 0.25 percentage point decrease in the share of automatable jobs overall, with a 0.55 percentage point decline being implied for manufacturing, and separately a 0.07 percentage point decline being implied for banking and finance. For workers aged between 26 and 39 the overall effect is centered around zero and not significant, however the estimates do imply that a minimum wage increase of 10% leads to a 0.1 percentage point decline in the share of automatable jobs available to these workers in manufacturing. For workers aged younger than 25 years, there are significant effects for the shares of employment analysis for the sub analysis of banking and finance as well as manufacturing. Specifically, the estimates imply that a minimum wage increase of 10% leads to a 0.06 and 0.08 percentage point decline in the share of automatable jobs available to these workers in these industries respectively.

**Table 3:** Shares of employment analysis.

	(1)	(2)	(3)	(4)	(5)	(6)	(7)	(8)
	Pooled	Manufacturing	Construction	Hotels and Restaurants	Transport and Communication	Banking and Finance	P Admin Educ and Health	Other Services
40 years old +								
Min Wage	-0.0251	-0.0549	-0.0007	-0.0026	-0.0022	-0.0074	-0.0014	-0.0011
	(0.0050)	(0.0130)	(0.0002)	(0.0008)	(0.0008)	(0.0026)	(0.0011)	(0.0003)
N	4320	480	480	480	480	480	480	480
26–39 Years Old								
	-0.0011	-0.0098	0.0020	0.0020	-0.0012	0.0002	-0.0003	0.0003
	(0.0004)	(0.0011)	(0.0004)	(0.0014)	(0.0006)	(0.0009)	(0.0011)	(0.0005)
N	4320	480	480	480	480	480	480	480
25 years or younger								
	-0.0044	-0.0064	-0.0002	-0.0002	-0.0018	-0.0075	-0.0004	-0.0004
	(0.0012)	(0.0014)	(0.0001)	(0.0003)	(0.0002)	(0.0026)	(0.0005)	(0.0002)
N	3263	426	383	431	383	442	443	480
Women								
Min Wage	-0.0053	-0.0180	-0.0008	-0.0026	0.0001	-0.0018	-0.0017	0.0000
	(0.0015)	(0.0017)	(0.0004)	(0.0015)	(0.0004)	(0.0016)	(0.0007)	(0.0003)
N	4320	480	480	480	480	480	480	480
Men								
Min Wage	-0.0177	-0.0390	-0.0011	-0.0025	-0.0012	-0.0047	-0.0035	-0.0018
	(0.0030)	(0.0055)	(0.0004)	(0.0028)	(0.0006)	(0.0021)	(0.0022)	(0.0007)
N	4320	480	480	480	480	480	480	427
White								
	-0.0184	-0.0299	-0.0013	-0.0006	-0.0011	-0.0056	-0.0020	-0.0018
	(0.0057)	(0.0070)	(0.0006)	(0.0024)	(0.0010)	(0.0029)	(0.0016)	(0.0014)
N	4320	480	480	480	480	480	480	480
Black								
Min Wage	-0.0004	0.0003	0.0001	-0.0008	0.0001	-0.0013	0.0003	-0.0000
	(0.0003)	(0.0004)	(0.0004)	(0.0004)	(0.0002)	(0.0004)	(0.0003)	(0.0002)
N	3612	452	440	451	445	455	459	443
Asian								
Min Wage	-0.0007	-0.0003	-0.0001	-0.0001	-0.0001	-0.0034	-0.0001	-0.0000
	(0.0004)	(0.0006)	(0.0006)	(0.0006)	(0.0007)	(0.0016)	(0.0005)	(0.0006)
N	3486	370	396	358	369	383	390	410

Notes: See Notes to Table 2

From Table 3, minimum wage changes predict more movement in the shares of automatable employment for men as compared with women. The estimates imply a minimum wage increase of 10% leads to a 0.18 percentage point decline in the share of automatable jobs for men versus 0.05 percentage points for women. Notably, the effects for manufacturing are again more substantive for both genders. For example, the share of automatable employment for men decreases by 0.39 percentage points in response to a 10% increase in the minimum wage. In comparison, for women, this fall is more modest at 0.18 percentage points. Table 3 also documents significant declines for men’s share of automatable employment in banking and finance.

Table 3 also disaggregates the estimates from Table 2 by ethnicity. For Whites, the significant pooled estimates are largely driven by changes to the share of low-skilled jobs that are automatable in manufacturing. For example, the pooled analysis implies that a 10% increase in the minimum wage leads to a 0.18 percentage point decline in the share of automatable employment, compared to 0.30 for manufacturing alone. The overall effect for the Asian and Black shares of employment analysis is centered around zero and not significant. Notably, the shares to be significantly affected for Asian and Black workers pertain to banking and finance. Specifically, the estimates in Table 3 imply that a 10% increase in the minimum wage increase causes a 0.03 and 0.01 percentage point decline in the share of automatable employment available for low skilled Asian and Black workers respectively.

The hypothesis of this work is that minimum wage increases accelerates a firm’s decision to automate tasks previously done by low-skilled low-wage individuals more quickly. A natural falsification for the shares of employment analysis emerges which re-calculates the shares of automatable employment for the highest-skilled group. I define the highest-skilled group as those with a university degree who work in occupations in the highest income quantile. Given my hypothesis I expect that minimum wage changes will have zero effect on the shares of employment of the highest skilled, or indeed positive effects if it is individuals of the highest skill that are hired as complements to the new technology. I document the results from these analyses in S1 File. I note that most estimates are centred around zero, with no coefficient that is statistically significant from zero.

### Proportion of workers paid less than the minimum wage:

This work relies on the QLFS, which consistently identifies twenty government regions across the period of analysis. It is reasonable to hypothesize that minimum wage increases can have varying effects depending on the proportion of the population affected by increases. Therefore, I also re-estimated Eq (3) and replaced the minimum wage variable with the proportion of workers paid less per hour than the minimum wage one year before the minimum wage was introduced in a particular area, as defined by hourly wage if reported, or wage per hour constructed from gross weekly wages divided by the number of usual weekly hours when not. This captures the proportion of workers who would expect to get a minimum wage increase in one year’s time if they remain employed. An advantage of this strategy is that it creates cross area variation, so I can replace the area specific time trends with area-specific year dummies, which is more conservative and more consistent with the approach taken to control variables by Lordan and Neumark [1]. The results for this analysis are provided in Table 4. Overall, there is concordance with what has gone before. That is, the overall effects on the shares of employment are modest, but there is evidence of more substantive changes in the shares of employment for manufacturing, men and older workers.

**Table 4:** Alternative minimum wage definition.

	(1)	(2)	(3)	(4)	(5)	(6)	(7)	(8)
	Pooled	Manufacturing	Construction	Hotels and Restaurants	Transport and Communication	Banking and Finance	P Admin Educ and Health	Other Services
Dependent Variable = Share of Automatable Employment								
Min Wage	-0.0212	-0.1923	-0.019	-0.0052	0.0001	-0.0241	-0.0048	-0.0131
	(0.0032)	(0.0114)	(0.0025)	(0.0028)	(0.0071)	(0.0041)	(0.0025)	(0.0039)
N	4320	480	480	480	480	480	480	480
	Men	Women	> = 40 years	<40 years&>25 years	<25 Years	White	Black	Asian
Min Wage	-0.0178	-0.0099	-0.0381	-0.040	-0.0154	-0.0099	-0.0005	-0.0024
	(0.0009)	(0.0025)	(0.0007)	(0.0010)	(0.0007)	(0.0039)	(0.0004)	(0.0011)
N	4320	4320	4320	4320	3263	4320	3612	3486

Notes: See Notes to Table 2

### Effects on remaining employed

The shares of employment analyses highlight the possibility that the absolute number of low skilled automatable jobs decreased in the last three decades in response to changes in the minimum wage. These effects are mostly concentrated in manufacturing. However, there are two reasons why I may get negative and significant effects in my shares of employment analysis. The first is job loss. However, it is also possible that the numerator is growing, implying that there are actually job gains in non-automatable low skilled jobs.

To consider whether a higher minimum wage actually increases dis-employment among low-skilled low-wage workers who were in automatable employment relative to workers in non-automatable work, Table 5 reports estimates of Eq (5), which models the effects of the minimum wage on the probability a particular individual who holds an automatable is still employed, as opposed to being dis-employed, compared to those in non-automatable work. Given the effect is identified from the interaction between minimum wages and being in automatable work, the baseline minimum wage effect is then not identified because of the inclusion of year*area fixed effects.

**Table 5:** Individual level estimates: Probability of being employed in the next period.

	(1)	(2)	(3)	(4)	(5)	(6)	(7)	(8)
	Pooled	Manufacturing	Construction	Hotels and Restaurants	Transport and Communication	Banking and Finance	P Admin Educ and Health	Other Services
Full Sample								
Min Wage	-0.0019	-0.0028	-0.0018	-0.0002	-0.0018	-0.0001	-0.0007	-0.0020
*Automatable	(0.0009)	(0.0016)	(0.0021)	(0.0015)	(0.0018)	(0.0015)	(0.0008)	(0.0018)
N	440614	75965	20858	58546	45190	60430	108347	37373
> = 40 years								
Min Wage	-0.0033	-0.0098	-0.0012	0.0004	-0.0006	-0.0021	0.0004	-0.0028
*Automatable	(0.0010)	(0.0032)	(0.0024)	(0.0020)	(0.0031)	(0.0023)	(0.0010)	(0.0025)
N	211312	40560	9636	24065	28711	22378	45870	13410
>25 years and <40 years								
Min Wage	-0.0010	-0.0015	-0.0025	0.0003	0.0010	-0.0008	-0.0013	-0.0004
*Automatable	(0.0004)	(0.0012)	(0.0021)	(0.0012)	(0.0017)	(0.0015)	(0.0010)	(0.0014)
N	196397	32436	9912	28804	15179	34140	52525	18041
<25 years								
Min Wage	-0.0010	-0.0072	0.0057	-0.0015	-0.0115	0.0005	0.0003	0.0054
*Automatable	(0.0050)	(0.0035)	(0.0124)	(0.0053)	(0.0098)	(0.0068)	(0.0054)	(0.0105)
N	32905	2969	1310	5677	1300	3912	9952	5922
Men								
Min Wage	-0.0045	-0.0082	-0.0019	-0.0024	-0.0010	-0.0030	-0.0032	-0.0059
*Automatable	(0.0015)	(0.0029)	(0.0017)	(0.0021)	(0.0025)	(0.0015)	(0.0020)	(0.0031)
N	249302	49097	15500	35345	29167	33387	35928	25611
Women								
Min Wage	-0.0023	-0.0044	-0.0022	-0.0009	-0.0014	-0.0023	0.0000	-0.0005
*Automatable	(0.0009)	(0.0031)	(0.0048)	(0.0018)	(0.0025)	(0.0024)	(0.0009)	(0.0020)
N	191312	26868	5358	23201	15993	27043	72419	11762
White								
Min Wage	-0.0015	-0.0077	-0.0001	0.0003	-0.0001	-0.0020	-0.0009	0.0016
*Automatable	(0.0004)	(0.0026)	(0.0017)	(0.0019)	(0.0019)	(0.0019)	(0.0007)	(0.0017)
N	71631	18622	51732	42758	55037	100398	30263	2787
Black								
Min Wage	-0.0028	-0.0095	0.0053	-0.0014	-0.0031	-0.0048	0.0057	0.0069
*Automatable	(0.0039)	(0.0081)	(0.0047)	(0.0025)	(0.0121)	(0.0069)	(0.0047)	(0.0074)
N	18921	2348	983	2897	854	2635	3488	3697
Asian								
Min Wage	-0.0017	-0.0002	0.0000	0.0056	-0.0042	-0.0040	0.0035	0.0000
*Automatable	(0.0067)	(0.0004)	(0.0000)	(0.0060)	(0.0048)	(0.0024)	(0.0040)	(0.0000)
N	20362	1986	1253	3917	1578	2758	4461	3413

Notes: OLS coefficient estimates are reported, with standard errors in parentheses. Standard errors are double clustered by area crossed by industry and year. Low-skilled workers are defined as those who have a GCSE equivalent or less and work in an occupation that is in the lowest quantile of the income distribution. The definition of automatable employment is created from variables in the UK Skills and Employment Surveys Series Dataset. A job is classified as automatable at the three-digit occupation code level. The share of automatable employment is calculated by industry, state, and year. All regressions include area crossed by year fixed effects. The pooled regression also has industry fixed effects. The minimum wage is measured in 2015 prices.

From Table 5 Column (1), I find evidence of significant declines in the probability of remaining employed in the next period–and hence being dis-employed–for those who were previously in automatable jobs as compared with those in non-automatable jobs. Specifically, the estimates imply declines in the probability of employment, from a 10% minimum wage increase, of 0.19 percentage points, with declines specific to manufacturing being 0.28 percentage points. The latter compares to a 0.48 percentage point decline found in Lordan and Neumark [1].

There is overall robustness in the estimated effects by sub-group, as compared with the shares of employment analyses. The most adverse employment effects are for the oldest (greater than 40 years) workers. Specifically, there are small but significant declines in the probability of employment of 0.03 percentage points for low-skilled workers in automatable employment, following a 10% increase in the minimum wage, who are > = 40 years as compared with comparable persons in non-automatable employment. The declines are again mainly driven by manufacturing (an implied decrease of 0.1 percentage points). Men in automatable work also experience a greater threat of dis-employment as compared to women, with significant declines in response to a 10% increase in the minimum wage in manufacturing, banking and finance and other services. Specifically, the declines expected are 0.08, 0.03 and 0.06 percentage points respectively. Looking at the effects by race, the pooled estimates are negative and significant for White workers, with these effects being driven mainly by declines in manufacturing. For example, a 10% increase in the minimum wage reduces by 0.08 percentage points the overall probability of remaining employed in the next period for White low skilled low wage workers in automatable employment. The effects for Black and Asian workers are noisy, but do suggest significant declines in banking and finance for Asian workers (0.04 percentage point decline).

### Hours effects

It is also possible that firms substitute with technology and decrease the hours of their employees, rather than culling their jobs I consider this explicitly by re-estimating Eq (4) and relating minimum wage variation to an alternate dependent variable. Here, the dependent variable is the share of hours worked among low-skill workers in automatable employment, in a particular industry, area, and year.

I also re-estimate Eq (7) with the difference in reported usual hours worked between this year and last year by an individual as the dependent variable. I focus only on those who are employed in the two periods (quarter 1 and quarter 5) and report non-zero working hours in both periods.

The results for the shares of hours analysis are reported in Table 6. The pooled estimates imply that minimum wage increases do decrease shares of hours for low-skilled low-wage workers in automatable employment significantly. For example, a 10% increase in the minimum wage causes a 0.04 percentage point decrease in the share of hours in automatable jobs done by low-skilled low-wage workers overall. The estimated declines in manufacturing are 0.19 percentage points. Consistent with the share of employment analysis, the share of hours analysis suggests that men are most affected at the intensive margins, along with the oldest and White workers.

**Table 6:** Hours based analysis.

	Pooled	Manufacturing	Construction	Hotels and Restaurants	Transport and Communication	Banking and Finance	P Admin Educ and Health	Other Services
Dependent Variable = Share of Automatable Hours								
Min Wage	-0.0037	-0.0187	0.0027	-0.0022	0.0013	-0.0009	-0.0034	0.0025	
	(0.0009)	(0.0021)	(0.0007)	(0.0039)	(0.0023)	(0.0043)	(0.0040)	(0.0015)	
N	4320	480	480	480	480	480	480	480	
	Men	Women	> = 40 years	<40 years&>25 years	<25 Years	White	Black	Asian	
Min Wage	-0.0049	0.0003	-0.0060	-0.0034***	-0.0020	-0.0041	-0.0007	0.0001	
	(0.0008)	(0.0005)	(0.0012)	(0.0008)	(0.0009)	(0.0010)	(0.0002)	(0.0002)	
N	4320	4320	4320	4320	3263	4320	3612	3486	
Dependent Variable = Difference in Hours between t and t+1 : Automatable Analysis								
	Pooled	Manufacturing	Construction	Hotels and Restaurants	Transport and Communication	Banking and Finance	P Admin Educ and Health	Other Services
Min Wage	-0.0843	-0.7993	-0.2683	0.0935	-0.2260	-0.1941	-0.1043	-0.2516
*Automatable	(0.0402)	(0.1637)	(0.2808)	(0.2646)	(0.2267)	(0.1381)	(0.1827)	(0.2531)
N	427309	69105	19483	56191	43886	58664	97180	36041
	Men	Women	> = 40 years	<40 years&>25 years	<25 Years	White	Black	Asian
Min Wage	-0.2044	0.1677	-0.1627	0.0390	0.2586	-0.0093	-0.2767	0.0518
*Automatable	(0.0634)	(0.1517)	(0.1843)	(0.1383)	(0.3347)	(0.0173)	(0.5117)	(0.0026)
N	4320	4320	4320	4320	3263	4320	3612	3486

Notes: See notes to Table 5.

The individual-level analysis considers the difference in the usual hours worked per week between quarter 1 and quarter 5. Based on the pooled estimate, a 10% increase in the minimum wage generates a 0.08 decrease in weekly hours worked for low-skilled individuals who held an automatable job in the previous period. In Table 6 the declines are only statistically significant in manufacturing for low-skilled low-wage individuals in automatable work. Turning to the sub analysis by gender, men again are more affected than women, but none of the other estimates are statistically significant. Overall the hours analysis reveals that there are effects at the intensive margin, in addition to the extensive margin.

### Effects on occupational switching

Table 7 reports results from an analysis where the dependent variable is equal to one if an individual stayed in the same occupation in the fifth quarter, and zero otherwise. Thesample includes all those employed in quarter 1 who have valid 3-digit occupation codes. Thus, the estimated effect of the minimum wage-routine interaction captures the change in job opportunities in the worker’s initial occupation, with a “decline” defined as either dis-employment *or* a change of job.

**Table 7:** Occupation stayers.

	(1)	(2)	(3)	(4)	(5)	(6)	(7)	(8)
	Pooled	Manufacturing	Construction	Hotels and Restaurants	Transport and Communication	Banking and Finance	P Admin Educ and Health	Other Services
Dependent Variable = Probability of Being employed in the Next Period: Automatable Analysis								
Min Wage	-0.0016	-0.0070	0.0000	0.0002	0.0000	-0.0065	0.0002	0.0000
*Automatable	(0.0009)	(0.0011)	(0.0004)	(0.0009)	(0.0003)	(0.0012)	(0.0005)	(0.0004)
N	400613	74192	19916	58401	44903	59846	99876	35417
	Men	Women	> = 40 years	<40 years&>25 years	<25 Years	White	Black	Asian
Min Wage	-0.0033	0.0010	-0.0020	-0.0051	-0.0006	-0.0012	-0.0029	0.0012
*Automatable	(0.0010)	(0.0007)	(0.0011)	(0.0015)	(0.0019)	(0.0009)	(0.0021)	(0.0014)
N	239607	161006	181099	195418	24096	366721	16018	17874

Notes: See notes to Table 5.

From Table 7 manufacturing and banking and finance have relatively comparable estimates, suggesting that higher minimum wages lead to some occupational switching among low-skilled low-wage workers in automatable jobs in these industries, in addition to transitions to dis-employment. Workers between the ages of 26 and 39 years have the largest effects, implying a greater propensity to switch because of automation. Given that the oldest workers were the most likely to lose their jobs in response to a minimum wage increase, this suggests middle-aged workers are more able to respond to higher minimum wages by getting an alternative occupation, but are suffering the switching costs. Low-skilled men in automatable work are more likely to switch jobs in response to a minimum wage increase, as compared with women. Finally, low-skilled Black workers are most likely to switch jobs in response to a minimum wage increase, suggesting that Black workers may also be more resilient to job loss with respect to job search.

### Contemporary analysis

The effects found so far are relatively modest and a question arises as to whether the effects are larger for a more recent sub-period, given that advances in technology moved forward with time and the associated cost fell. To explore this, similar to Lordan and Neumark [1] I re-estimate Eq 3 for the share of employment analysis and Eq 5 for the probability of remaining employed analysis for the period 2004 to 2017. The estimates are documented in Table 8 and it is notable that they are roughly double the size of those documented in Tables 2, 3 and 5 for sub-groups where significant effects were originally found, namely manufacturing, men, older and White workers. For example, for manufacturing, the shares of employment analysis in Table 4 suggest that a minimum wage increase of 10% leads to a 0.47 percentage point decrease in the share of automatable jobs done by low-skilled low -wage workers in manufacturing for the period 2004 to 2017. This compares to 0.28 in Table 2.

**Table 8:** Contemporary analysis.

	(1)	(2)	(3)	(4)	(5)	(6)	(7)	(8)
	Pooled	Manufacturing	Construction	Hotels and Restaurants	Transport and Communication	Banking and Finance	P Admin Educ and Health	Other Services
Dependent Variable = Share of Employment								
Min Wage	-0.0131	-0.0471	-0.0001	-0.0049	0.0030	-0.0069	-0.0094	-0.0056
	(0.0039)	(0.0043)	(0.0012)	(0.0020)	(0.0038)	(0.0042)	(0.0040)	(0.0016)
N	2340	260	260	260	260	260	260	260
	Men	Women	> = 40 years	<40 years&>25 years	<25 Years	White	Black	Asian
Min Wage	-0.0293	-0.0018	-0.0375	-0.0049	-0.0024	-0.0308	-0.0066	-0.0000
	(0.0042)	(0.0011)	(0.0055)	(0.0023)	(0.0007)	(0.0051)	(0.0022)	(0.0001)
N	2340	2340	2340	2340	2340	2321	1150	793
Dependent Variable = Probability of Being Unemployed								
	Pooled	Manufacturing	Construction	Hotels and Restaurants	Transport and Communication	Banking and Finance	P Admin Educ and Health	Other Services
Min Wage	-0.0035	-0.0061	0.0035	-0.0022	-0.0026	-0.0044	0.0007	-0.0026
*Automatable	(0.0019)	(0.0027)	(0.0029)	(0.0018)	(0.0019)	(0.0025)	(0.0010)	(0.0022)
N	256411	39918	11415	28651	25009	32712	52196	20721
	Men	Women	> = 40 years	<40 years&>25 years	<25 Years	White	Black	Asian
Min Wage	-0.0026	-0.0006	-0.0043	-0.0029	-0.0022	-0.0036	-0.0033	-0.0005
*Automatable	(0.0012)	(0.0008)	(0.0025)	(0.0013)	(0.0012)	(0.0019)	(0.0039)	(0.0066)
N	154870	10541	133437	100908	22066	38484	10269	12455

Notes: See notes to Table 2 and Table 8

In addition, the estimates in the shares of employment analysis are significant and negative, albeit modest, for hotels and restaurants, banking and finance, public administration, education and health and other services. Overall, Table 8 suggests that the average effects calculated for the period 1994–2017 mask larger effects that occurred over the last decade and a half.

## Conclusions

In 2020 the minimum wage will be 60 per cent of median hourly earnings, around £8.75 in the UK, after experiencing continuous annual increases since its introduction in 1999. Given this trend, this study explores whether these previous minimum wage increases have affected the employment possibilities for low skilled low wage workers relying on automatable employment, following Lordan and Neumark [1]. Drawing on the QLFS from 1994–2017 and classifying each occupation as either automatable or non-automatable I consistently highlight that minimum wage increases significantly decrease the shares of automatable employment available to low-skilled low-wage workers following a minimum wage increase. However, these effects are modest in size. For example, an increase of 10% in the minimum wage implies a 0.11 percentage point decrease in the shares of automatable employment. This effect is about one third the size found by Lordan and Neumark [1] for the US. However, these aggregate effects mask larger changes for manufacturing, older workers, men and White workers. In addition, I also present estimates for 2004–2017 which illustrate an effect that is roughly double in size. This suggest that the interaction between the minimum wage and automation has been accelerating as technology advanced, its price falls and the minimum wage increases.

A consistent narrative also emerges for an analysis which considers the likelihood a low-skilled low-wage worker in automatable employment remains employed in the next year, as compared to a comparable worker in non-automatable employment following a minimum wage increase. Specifically, while aggregate effects are modest the sub-analysis reveals that workers in manufacturing are the most vulnerable to the loss of automatable work, as well as low skilled men, White and older workers. I also highlight that the same types of low-skilled low-wage workers in automatable employment are more likely to switch jobs in the next period following a minimum wage increase. An analysis of hours also reveals that there are effects at the intensive margins. In addition, an analysis from 2004–2017 reveals estimates that are substantively larger, once again suggesting that the average estimates I have identified between 1994–2017 were increasing over time.

Overall, this study suggests that firms may re-assess their production processes following minimum wage increases. Given that the analysis for a more recent sub period (2004–2017) suggests that the effects found are increasing over time I emphasis the importance of taking the potential for automation to interact with the minimum wage seriously. Given that the costs of technology continue to fall, and many more low skill jobs are on stream to be automated in the future (including security guard, driver shelf stacker and brick layer), while the effects found in this study are modest, they should not be used to predict the future. Rather, monitoring of these trends, and ensuring that low skilled low wage individuals are not unduly hurt by the advent of the 4^th^ industrial revolution is a key role for government and social science researchers.

## Supporting information

S1 File (PDF)
