# Supplementary material for: People versus machines in the UK: Minimum wages, labor reallocation and automatable jobs
Source: PLoS One. 2019 Dec 2;14(12):e0224789. doi: 10.1371/journal.pone.0224789 (PMC6886789; doi:10.1371/journal.pone.0224789)
Supplement: S1 File — (PDF) [file pone.0224789.s001.pdf]

Supporting Information

S1 Appendix

S1 Table: Pooled Analysis

	(1)	(2)	(3)	(4)	(5)	(6)	(7)	(8)
	Pooled	Manufacturing	Construction	Hotels and Restaurants	Transport and Communication	Banking and Finance	Public Admin Education and Health	Other Services
Dependent Variable = Share of Automatable Employment								
Min Wage	0.0002	0.0005	0.0000	-0.0002	0.0000	-0.0000	0.0014	0.0000
	(0.0005)	(0.0004)	(0.0001)	(0.0002)	(0.0002)	(0.0001)	(0.0008)	(0.0002)
N	4320	480	480	480	480	480	480	480

Notes: OLS coefficient estimates are reported, with standard errors in parentheses. Min Wage is in log form. Standard errors are double clustered by area crossed by industry and year. I define the highest-skilled group as those with a university degree who work in occupations in the highest income quantile. The definition of automatable employment follows Autor and Dorn (2013) and Autor et al. (2015) and is consistent with that used by Lordan and Neumark (2018). A job is classified as automatable at the three-digit occupation code level. The share of automatable employment is calculated by industry, state, and year. All regressions include area fixed effects and area specific time trends. Regressions also include other control variables. These are: 1) Lag of Area level unemployment rate. 2) Lag of Industry level unemployment rate. 3) Lagged Area level demographics that vary over time: average age, education, gender. 4) Occupation demographics measured at the area/industry/year level. Specifically, these are average age, education, gender. All lagged variables relate to one year. We note that the estimates do not change notably (to the third decimal place) if lags are replaced with contemporaneous values.
